# 13q deletion syndrome resulting from balanced chromosomal rearrangement in father: the significance of parental karyotyping

**DOI:** 10.1186/s13039-020-00500-7

**Published:** 2020-07-23

**Authors:** Sabine Dittner-Moormann, Madlen Reschke, Eva Biewald, Alma Kuechler, Barbara Klein, Beate Timmermann, Dietmar Lohmann, Petra Ketteler, Deniz Kanber

**Affiliations:** 1 Department of Pediatric Hematology and Oncology, University Hospital Essen, Hufelandstraße 55, 45147 Essen, Germany; 2 Department of Pediatric Hematology, Oncology and Stem Cell Transplantation, Charité - Universitätsmedizin Berlin, Berlin, Germany; 3 Department of Ophthalmology, University Hospital Essen, Essen, Germany; 4 Institute of Human Genetics, University Hospital Essen, Essen, Germany; 5 Sozialpädiatrisches Zentrum, Klinikum Frankfurt Höchst GmbH, Frankfurt a. M, Germany; 6 Department of Particle Therapy, West German Proton Therapy Centre Essen (WPE), West German Cancer Center (WTZ), German Cancer Consortium (DKTK), University Hospital Essen, Essen, Germany

**Keywords:** Retinoblastoma, 13q deletion, Complex chromosomal rearrangement, Balanced translocation

## Abstract

**Background:**

Retinoblastoma is a malignancy of the eye in children characterized by biallelic inactivation of *the retinoblastoma 1* gene (*RB1*), located at chromosome 13q14.2. Children with interstitial chromosome 13q deletions that include the *RB1* gene show a predisposition to develop retinoblastoma and variable other features. Large 13q deletions with severe clinical phenotype are nearly always the result of a de novo mutation, i.e. the pathogenic alteration is not detected in parents. This results in a low risk for siblings to develop 13q deletion syndrome.

**Result:**

Here, we describe a patient with profound muscle hypotonia, severe developmental delay and bilateral retinoblastoma carrying a large deletion in 13q13.3q14 with the size of 16 Mb, involving the *RB1* gene. Neither parent showed retinoblastoma, muscle hypotonia or developmental delay. Chromosome analysis and Fluorescence in situ hybridization (FISH) showed a balanced complex chromosomal rearrangement (CCR) between chromosome 12 and 13 [ins(12;13)(q21.2;q12.3q14.3)] and an additional balanced translocation of chromosome 7 and 15 [t(7;15)(q31.2;q25.3)] in the healthy father. Malsegregation of the paternal insertional translocation involving chromosome 12 and 13 resulted in a 13q deletion syndrome of the child [46,XY,ins(12;13)(q21.2;q12.3q14.3)].

**Conclusion:**

Balanced translocations in parents are a rare cause of de novo *RB1* deletions in offspring. This case report emphasizes the need for parental chromosomal analysis and FISH in parents of children diagnosed with 13q deletion syndrome or large *RB1* gene deletions to precisely determine the recurrence risk in siblings. Guidelines for genetic testing should be revised accordingly.

## Background

Retinoblastoma (Rb) is a rare cancer, but the most common malignant neoplasm of the eye in children. The estimated global incidence is 1/15,000 to 1/20,000 live births [1]. In about 60% of all patients with Rb the tumor affects only one eye (unilateral Rb), usually with a single tumor focus. Both eyes are affected in 40% of patients with Rb (bilateral Rb), mostly with multiple tumor foci. Rb occurs almost exclusively in children under the age of 5 years. In Germany, the median age at diagnosis is 24 months for the unilateral, unifocal type and < 12 months for bilateral Rb [2].

Rb arises from retinal cone cell progenitors that have lost normal function of the retinoblastoma protein (pRb) as a consequence of genetic alterations of both alleles of the *RB1* gene. Patients with heritable Rb are heterozygous for a variant allele of the *RB1* gene (first mutation) and usually develop Rb in both eyes (bilateral Rb). In most patients with bilateral Rb this variant allele is the result of a de novo mutation in the germline of a parent. Development of Rb is triggered by a second mutational event that occurs in somatic cells and results in alteration or loss of the other allele. Patients with heritable Rb have a high risk of developing further non ophthalmologic tumors [3]. In non-heritable Rb presenting mostly as unilateral unifocal tumor both *RB1* alleles are altered as result of mutations in somatic cells.

Patients with deletions on chromosome 13q show variable clinical features and a high risk of Rb if the *RB1* gene is involved. Chromosome 13 is the largest acrocentric human chromosome, bearing one of the lowest gene densities (6.5 genes per Mb) [4]. The association of mental and growth retardation with Rb was first described as an entity in 1969 [5]. Additional characteristics in patients with 13q deletion syndrome include moderate to severe mental retardation or psychomotor delay, muscular hypotonia, seizures, growth delay, craniofacial dysmorphic features and various congenital defects of the brain, eye, gastrointestinal tract, urogenital tract, kidney and heart [6, 7]. Size and location of chromosome 13q deletion affects clinical phenotype and the occurrence of Rb [7, 8]. Deletion of a chromosomal segment can occur de novo or can be inherited due to parental chromosomal aberration [7, 9].

## Material and methods

### Methylation-specific multiplex ligation-dependent probe amplification (MS-MLPA)

For methylation and dosage analysis of the *RB1* region MS-MLPA (MRC Holland, SALSA MLPA probemix P047-D1 RB1) was performed according to the manufacturer’s protocol.

### Cytogenetic and molecular cytogenetic analysis

Cytogenetic analysis was performed on GTG banded metaphase chromosomes from PHA stimulated blood lymphocytes [10]. The same metaphase preparations were also used for molecular cytogenetic analysis (FISH) which was performed according to standard procedures based on the manufacturer’s protocol. Following commercially available probes were used: whole chromosome painting probe wcp 12, MetaSystems, XCP12 Orange, Cat.No. D-0312-050-OR; wcp 13, MetaSystems, XCP13 Green, Cat.No. D-0313-050-FI; RB1, Abbott, Vysis LSI 13 RB1 (13q14) SpectrumOrange Probe, Cat.No. 08 L65–020. Images were captured on Zeiss Axioplan microscope (Zeiss Jena, Germany) with IKAROS and ISIS digital fluorescence in situ hybridization (FISH) imaging system (MetaSystems, Altlussheim, Germany).

## Case presentation

### Clinical report

The male patient was born spontaneously at 38 + 5 weeks of gestation after an uneventful pregnancy to healthy nonconsanguineous parents with unremarkable family history. Birthweight was 2610 g (3rd percentile, − 1.9 SDS), length 48 cm (5th percentile, − 1.6 SDS), head circumference 35 cm (44th percentile, − 0.14 SDS) [11], Apgar score 10/10. Muscular hypotonia was noticed postpartum. Cerebral ultrasound at 6 days of life showed slightly asymmetric lateral ventricles (right > left) but otherwise normal results. Screening by automated auditory brainstem response was normal. The patient showed craniofacial dysmorphism including a high forehead and deep-set ears. Developmental delay of motor function and speech were first diagnosed at the age of 11 months. Electroencephalography showed inconspicuous results. At the age of 22 months motor skills equaled those of a 7 month old. At the age of 3 years, the patient had made slight progress on his motor skills but not on language development.

Examination of the cardiovascular system including electrocardiogram and echocardiography showed normal results.

At the age of 9 months the parents observed leukocoria. At the age of 1 year bilateral Rb was diagnosed according to international classification of retinoblastoma (ICRB) and international retinoblastoma staging system (IRSS). The right eye was classified as ICRB E, IRSS I and the left eye as ICRB D, IRSS 0. The right eye was enucleated due to advanced tumor stage with signs for infiltration of the optic nerve on MRI scan. Histopathological examination of the enucleated right eye confirmed focal choroidal and intralaminar optic nerve infiltration.

The intensive eye-preserving treatment using chemotherapy and percutaneous proton beam radiotherapy for the remaining eye failed after multiple relapses. The second eye had to be enucleated 6 months after diagnosis. At that time ophthalmologic examination revealed no light perception, vision non lux. Histopathological examination of the eye showed neither choroidal, scleral nor optic nerve infiltration nor infiltration of the anterior segment.

During chemotherapy, the patient developed mild hearing impairment of the right ear (about 30 dB at 2–4 kHz).

### Genetic testing

After counselling and obtaining informed consent from the parents, molecular genetic testing was performed. Testing started with Multiplex Ligation-dependent Probe Amplification (MLPA) on DNA from blood, because syndromic features of the patient suggested a contiguous gene deletion syndrome including the *RB1* gene. Results of MLPA which included analysis of dosage and methylation showed a hemizygous deletion of the 13q13.3q14.3 region (16.6 Mb) including the *RB1* gene. Hypermethylation at CpG85 on the remaining allele, a characteristic feature of the maternal allele, suggested that the deleted allele is of paternal origin.

Paternal chromosomal analysis revealed balanced complex chromosomal rearrangement with a reciprocal translocation between long arms of chromosome 7 and 15 with breakpoints located at 7q31.2 and 15q25.3 and an insertional translocation involving chromosome 12 and 13 [46,XY,ins(12;13)(q21.2;q12.3q14.3)].

**Fig. 1:**
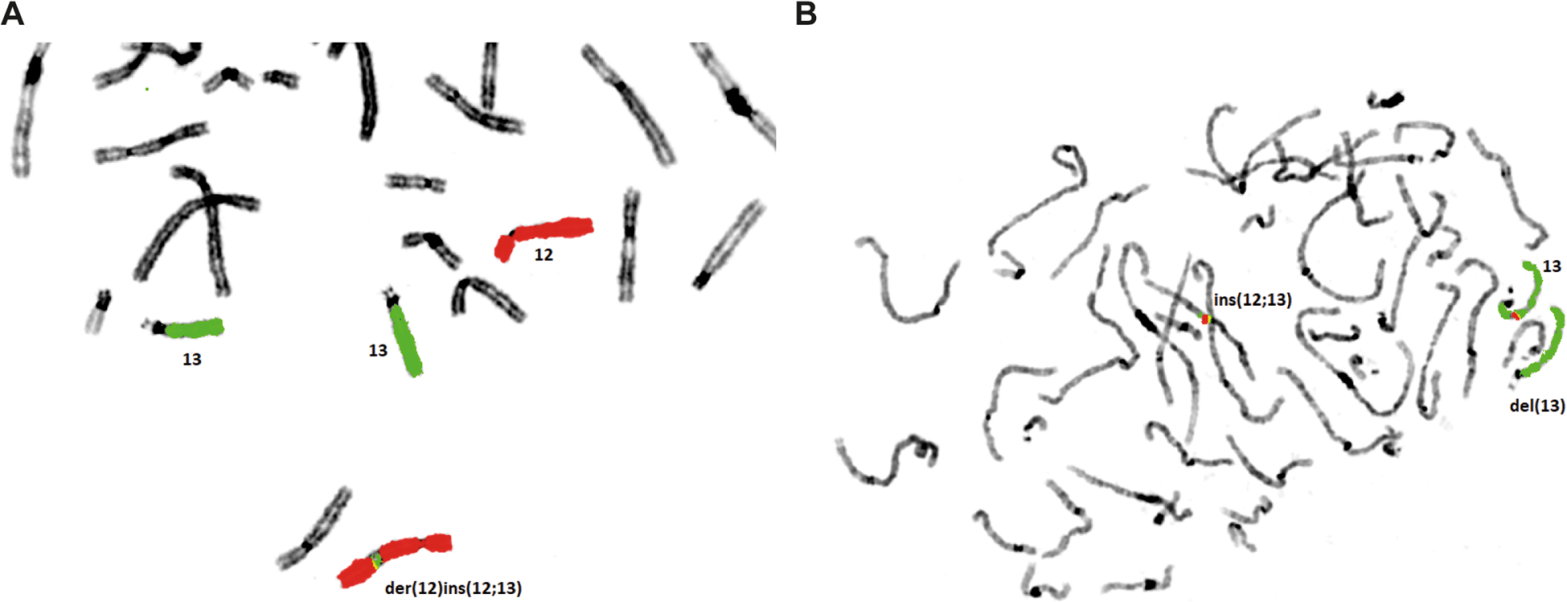
Balanced complex chromosomal rearrangement between chromosome 12 and 13 detected by FISH analysis on metaphase chromosomes of the patient’s father. **a** FISH with whole chromosome paint (wcp) probes for chromosome 12 (red) and 13 (green) reveals a signal for the probe wcp 13 (green) on one chromosome 12. Thus, the derivative chromosome 12 has an insertion of material from chromosome 13 (der(12)ins(12;13)). **b** The inserted Region contains the *RB1* gene. The probe for *RB1* (red) provides a signal on the derivative chromosome 12 and a signal on one of the chromosomes 13 (wcp 13, green) – no signal for *RB1* on del(13)

Aberration of chromosome 12 and 13 is an insertion of a short segment of chromosome 13 into the long arm of chromosome 12 at 12q21.2. This inserted fragment, 13q12.3q14.3, contained the *RB1*-locus as confirmed by FISH analysis (Fig. 1).

## Discussion and conclusion

This case report describes a patient with heritable retinoblastoma predisposition caused by a large deletion on chromosome 13q that arose on the basis of a balanced chromosomal rearrangement in the father.

Most 13q deletion syndromes are reported to occur de novo and, given the paucity of published cases it appears that cases similar to the one reported here are rare [9, 12, 13]. Strong et al. characterized a family in which 10 cases of unilateral Rb occurred over four generations transmitted by eight unaffected individuals. Chromosomal analysis of four living Rb patients revealed an interstitial deletion of the long arm of chromosome 13, del(13)(q13.1q14.5). Clinical characteristics of all Rb patients in the family included failure to thrive, developmental delay and mild to severe mental retardation. Retrospectively, these symptoms would be classified as part of 13q deletion syndrome. Chromosome analysis of all transmitting family members who were alive showed balanced karyotype with insertional translocation of the deleted chromosome 13 into the short arm of chromosome 13. Further eight individuals in this family showed an unbalanced triplication of the 13q(q13.1q14.5) segment, but this alteration was not associated with any specific clinical phenotype [14].

Another reported case of deletion of a 13q segment derived from a familial 12;13 insertional translocation. The phenotype was concordant with that expected of an interstitial deletion including the *RB1* locus: bilateral Rb, severe psychomotor retardation, failure to thrive, dysmorphic facial features, anomalous pinnae, accessory nipples, cryptorchidism, abnormal foot posturing and diffuse myocardial hypertrophy with disrupted fibre muscle orientation. Mother and grandmother carried the balanced insertional translocation. In one sibling of the patient the 13q chromosomal segment was triplicated. In line with the report of Strong et al. the phenotypic consequences appeared to be relatively modest [15].

Current suggestions for best practice of genetic testing in unaffected parents of patients with large *RB1* gene deletions include deletion testing but do not require chromosome analysis or other methods to detect balanced alterations in patient and parents [16]. This recommendation reflects that at least among published findings balanced alterations in a parent appear to be a rare cause of *RB1* gene deletions in children. Both previous reports [14, 15] were published at a time when cytogenetic analysis was the first line analysis for genetic testing in retinoblastoma. Today, molecular methods of deletion testing are favored over cytogenetic analysis. Moleculargenetic methods are not expected to detect balanced translocations and this may lead to an underestimation of the frequency of such alterations.

Genetic testing in parents is frequently motivated by the question of recurrence risk in future siblings. Current molecular methods such as MLPA will detect heterozygous or high level mosaic deletions, but balanced parental chromosomal alterations escape detection by molecular methods such as MLPA or array analysis. However, balanced alterations result in recurrence risk for siblings of up to 50%. The frequencies of submicroscopic insertional translocations as cause of apparent de novo chromosomal aberrations has been reported as high as 2% [17]. This reported frequency and the high recurrence risk in siblings warrants parental chromosome and FISH analysis as part of the clinical routine testing in all families with interstitial aberrations and therefore also in parents of children with 13q deletion syndrome. Detection of potential progenitors of *RB1* gene deletions are important for genetic counselling of the family and may warrant testing of further family members. On the ground of the presented findings cytogenetic analysis including FISH for the *RB1* locus in children with 13q deletion syndrome and in their parents are strongly recommended along with molecular genetic array diagnostics of large chromosomal aberrations. Only the combination of molecular and cytogenetic testing will provide high quality of genetic counselling and avoids the case of a present balanced chromosomal alteration in a parent being undetected.

## Data Availability

The datasets used and/or analyzed during the current study are available from the corresponding author on reasonable request.

## Abbreviations

CCR: Complex chromosomal rearrangement
FISH: Fluorescence in situ hybridization
ICRB: International classification of retinoblastoma
IRSS: International retinoblastoma staging system
MLPA: Multiplex Ligation-dependent Probe Amplification
MRI: Magnetic Resonance Imaging
Rb: Retinoblastoma
*RB1*: Retinoblastoma 1 gene
